# Exploring the education in cultural competence and transcultural care in Spanish for nurses and future nurses: a scoping review and gap analysis

**DOI:** 10.1186/s12912-023-01483-7

**Published:** 2023-09-16

**Authors:** Yasmin El-Messoudi, Manuel Lillo-Crespo, Juan Leyva-Moral

**Affiliations:** 1 Facultad de Ciencias de La Salud, Universidad de Málaga, Arquitecto Francisco Peñalosa, 3, 29071 Málaga, Spain; 2 Nursing Department, Faculty of Health Sciences, University of Alicante, Alicante, Spain; 3 Nursing Department, Faculty of Medicine, Universitat Autònoma de Barcelona, Barcelona, Spain

**Keywords:** Cultural competency, Transcultural nursing, Nursing education, University, Spain, Latin America, Review literature, Gray literature

## Abstract

**Background:**

Globalization and population migration have led to increasingly culturally diverse societies, which has made nursing education in cultural competence and transcultural care a priority. This includes the ability to provide person-centered and culturally congruent care, even within one's own culture. However, this sort of training has been developed and implemented in practice comparatively more by English-speaking societies. Therefore, the aim of this study was to identify the existing educational initiatives for nurses and future ones in cultural competence and transcultural care in Spanish and explore their didactic characteristics in terms of teaching and learning formats, contents, skills, and evaluation methods at different academic levels.

**Methods:**

A scoping review was carried out by following the specific PRISMA recommendations and those of the Joanna Briggs Institute guidance throughout PudMed, Web of Science, Embase, Google Scholar, and Cinahl databases and also gray literature in the form of official documentation that later was complemented with a gap analysis including training programs published by Spanish and Latin-American educational institutions and the approaches of key academic informants.

**Results:**

The published evidence on nursing training in cultural competence or related topics in Spanish-speaking higher education for nurses is limited. Specific nursing programs in Spanish-speaking universities are primarily found in Spain, with fewer options available in Latin America. These contents are offered either as optional subjects or immersed in other courses and mainly taught in theoretical sessions. Practice in real contexts is supposed to be evaluated transversally under the cultural scope according to national educational recommendations though barely visible in students’ evaluation reports. Even though postgraduate training dedicated to these issues exists, it is still limited, mixed with other contents, and generally depends on a few researchers investigating and publishing on the topic from very specific universities.

**Conclusions:**

It is essential to establish a common global strategy including Spanish-speaking countries in nurses’ higher education and professional training on topics focused on cultural competence as well as the provision of nurses’ social and cultural sensitivity towards their own culture and to define whether those that currently exist are effective. It is also crucial that this training was evaluated in practice in order to achieve enough impact on students, health organizations, and population health.

**Supplementary Information:**

The online version contains supplementary material available at 10.1186/s12912-023-01483-7.

## Introduction

Globalization and migration have produced an increase in cultural diversity in different societies worldwide [1]. According to the International Organization for Migration (IOM), Spain was in the sixth position in 2019 among the European countries with the highest number of people arriving from other nations, registering approximately 6 million foreigners living in the country at that time [2]. In Latin America, the number reached 14.8 million international migrants in 2020 [3]. These migratory flows have produced an increase in multiculturalism within all societies and therefore it has made health professionals face new cultural needs towards offering their patients quality care [4, 5]. This new situation might become a good opportunity for health professionals to also reflect on the recognition of their own culture, social aspects, and cultural self-awareness. In this way, education in cultural competence and transcultural nursing becomes a priority in order to provide individualized and appropriate care to all people [6].

The nursing profession involves caring for patients’ cultural needs, equitable access to health services, respecting their cultural values, and beliefs, and safeguarding those individuals’ needs [7]. Therefore it is essential to have a global cultural nursing perspective towards adapting the cultural differences and similarities of other groups and recognizing their own ones in order to integrate them into national health programs [8]. However, there is a lack of protocols and implementation plans regarding cultural knowledge, sensitivity, awareness, and competence in most countries even in the geographic areas where multiculturalism has been historically present. It is worth noting at this point that the term culture has to do not only with ethnicity, culturally diverse groups, and religious beliefs but also with day-to-day aspects that occur in different settings, systems, and organizational structures, gender, ways of dressing, gestures, cooking, the meaning of food, the perceptions of reality, thoughts, behaviors, attitudes among others [9].

Due to the complexity of the current societies, where migratory movements generate constant transformations in human interactions, cultural competence has become one of the most important strategic trends of research and education in nursing [10]. However, it has not always been developed equally in all societies and is even sometimes less promoted and barely recognized. Several authors have stressed that nurses need to have sufficient knowledge and training to be able to provide culturally competent care [1, 8, 11] though its implementation differs from one place to another.

## Background

Cultural competence as a concept was introduced by North American intercultural nursing theories, starting with the model developed by Madeleine Leininger in the 1970s [12]. According to Leininger, Cultural Care comprises the multiple aspects of culture that influence and help a person or group to improve their human condition or deal with illness or death, and therefore cultural competence is the skill to provide such care [13]. The American Academy of Nursing also defined cultural competence as the acquisition of knowledge, understanding, and skills about different cultural groups that allow the health professional to provide culturally acceptable care [14]. Over the years, the concept of cultural competence and transcultural care gained greater importance, especially in the United States (US) as one of the highest multicultural societies in the world. Cultural competence has become more relevant in the scientific community thanks to the emerging theories and the development boosted by the Transcultural Nursing Society (TCNS), with initiatives such as the specific Certification in Transcultural Nursing (CTN) recognized by the American Nursing Credentialing Center (ANCC) and therefore by most of the health organizations in the US as well as the amounts of scientific manuscripts with evidences and theories published in their Journal of Transcultural Nursing (JTCN) and other similar journals [15].

There are several authors who have created models related to cultural competence and transcultural care such as Larry Purnell and his model on cultural competence and skill acquisition [16], Giger and Davidhizar's model of transcultural nursing [17], and Spector's model of health traditions [18], Similarly, Campinha-Bacote developed a model that establishes that cultural competence is a process that nurses need to go through in order to provide high-quality and efficient care [19]. However, all of them belong to the US and have implemented research and education mainly in their country for years, having later expanded firstly through other English-speaking nations such as the United Kingdom (UK) and Australia. In Europe, Irena Papadopoulos developed a model for the development of cultural competence in nursing which highlights the importance of performing compassionate care [20], and much later arrived in the Spanish-speaking countries. Nowadays, the European Transcultural Nursing Association (ETNA) is a young association that includes a representation of several European countries, including Spain, though its impact on the European health systems and educational programs is far from the one achieved by the TCNS in the US [21].

If nurses achieve a high level of cultural competence, they will be able to maintain more effective transcultural communication, generating a better evaluation of the needs of patients, which will produce appropriate and compassionate care. This can lead to reducing inequalities in health care and improve the health outcomes of patients aftercare [22]. Cultural competence makes health systems work better and It improves and increases patients´satisfaction [23]. Thus, it is essential that decision-makers and educational organizations take into account the importance of adequate training in cultural competence for the appropriate approach to patients [24]. It has been shown in studies carried out in different countries of the world that health outcomes improve when nurses have an adequate level of cultural competence, although this level remains low and moderate [25–28]. This level of cultural competence stays that way especially in those countries where the importance of cultural competence is unseen or definitely has not been explored until now such as the Spanish-speaking ones [29].

Despite the fact that in Spain cultural competence constitutes a transversal competence in university educational programs [29], a study carried out by Plaza del Pino shows that there is a small nursing professional sample that considers it relevant [30]. University training on cultural diversity within the Nursing undergraduate program is scarce in contents and European Credit Transfers (ECTS), and sometimes is not part of the contents within the study programs [31]. Moreover, in Latin America, multiculturality has not yet been given sufficient importance in the Nursing educational system, despite the fact that laws have been developed and projects have been proposed aimed at improving practices for its respect [32]. It is also important to point out the misunderstanding of the different concepts and terms regarding this topic among health professionals in Spanish-speaking countries. The term "interculturality" refers to the interaction and therefore, mutual enrichment between different cultures while the concept of “multiculturalism” refers only to the presence of several cultures, without delving into anything else, that is, without exchange among different cultures [33]. On the other hand, Herskovits defined “transculturality” as those phenomena that result when groups of individuals, having different cultures, make continuous contact, with consequent changes in the patterns of the original culture of one of the groups or both [34].

In this regard, it is essential to develop a common strategy for higher education in Spanish-speaking countries in terms of cultural competence and transcultural care. However, published precedents show the need to reinforce the acquisition of skills in cultural competence of future nursing professionals, especially in clinical practice, since when they care for populations they become the main promoters of the right to health [35, 36]. Therefore, this study aims to examine how higher education institutions (HEIs) in Spanish-speaking countries promote and structure the training of nursing students and professionals in cultural competence, transcultural care, and related subjects. This includes undergraduate and postgraduate programs, as well as continuing professional development initiatives.

## Methods

The methodology selected is a scoping review for filling the gap of knowledge and mapping the situation in Spanish-speaking countries. The PRISMA Extension for scoping reviews (PRISMA-ScR) recommendations [37] were followed as well as those by the Joanna Briggs Institute guidance [38]. Scoping reviews are especially useful for addressing heterogeneous or broad issues where there are gaps of knowledge or a lack of consensus. Scoping reviews are a type of review that usually includes gray literature and therefore fits well with our study purpose. The available scientific literature and documentation such as national white papers and government reports from official institutions were explored. Then a gap analysis was conducted by consulting all the available academic study programs from Spanish and Latin-American HEIs, other related organizations, and the approaches of key academic informants to determine in which of them cultural competence, transcultural care, or other similar terms are studied and the characteristics of these programs.

### Search strategy

A preliminary peer-reviewed search was carried out to identify publications about the topic in evidence-based databases. The search was performed in Joanna Briggs Institute Evidence Synthesis, MEDLINE, as well as in the Cochrane Library by using intuitive terms such as “cultural competence”, “transcultural nursing”, “education”, “universities'', “Spain” and “Latin America” in English and Spanish; and the lack of publications regarding the topic was evident. Subsequently, another peer-reviewed search was performed through PubMed to identify the Mesh terms used towards establishing the keywords and developing the search strategy. The keywords used were firstly according to MESH terms (“Cultural competency”, “Nursing education”, “Transcultural nursing”, “Nursing students'', “Universities”, “Spain”, and “Latin America”) and later other synonyms were extracted and also added such as: “higher education”, “college”, “cultural nursing education”, “cultural competence” and “practical training”. This search strategy was adapted to each database (Additional file 1). The search took place between the months of February and December 2022 and included studies published in both English and Spanish from 2017 until 2022 throughout PudMed, Web of Science (WOS), Embase, CINAHL, and Google Scholar.

**Fig. 1:**
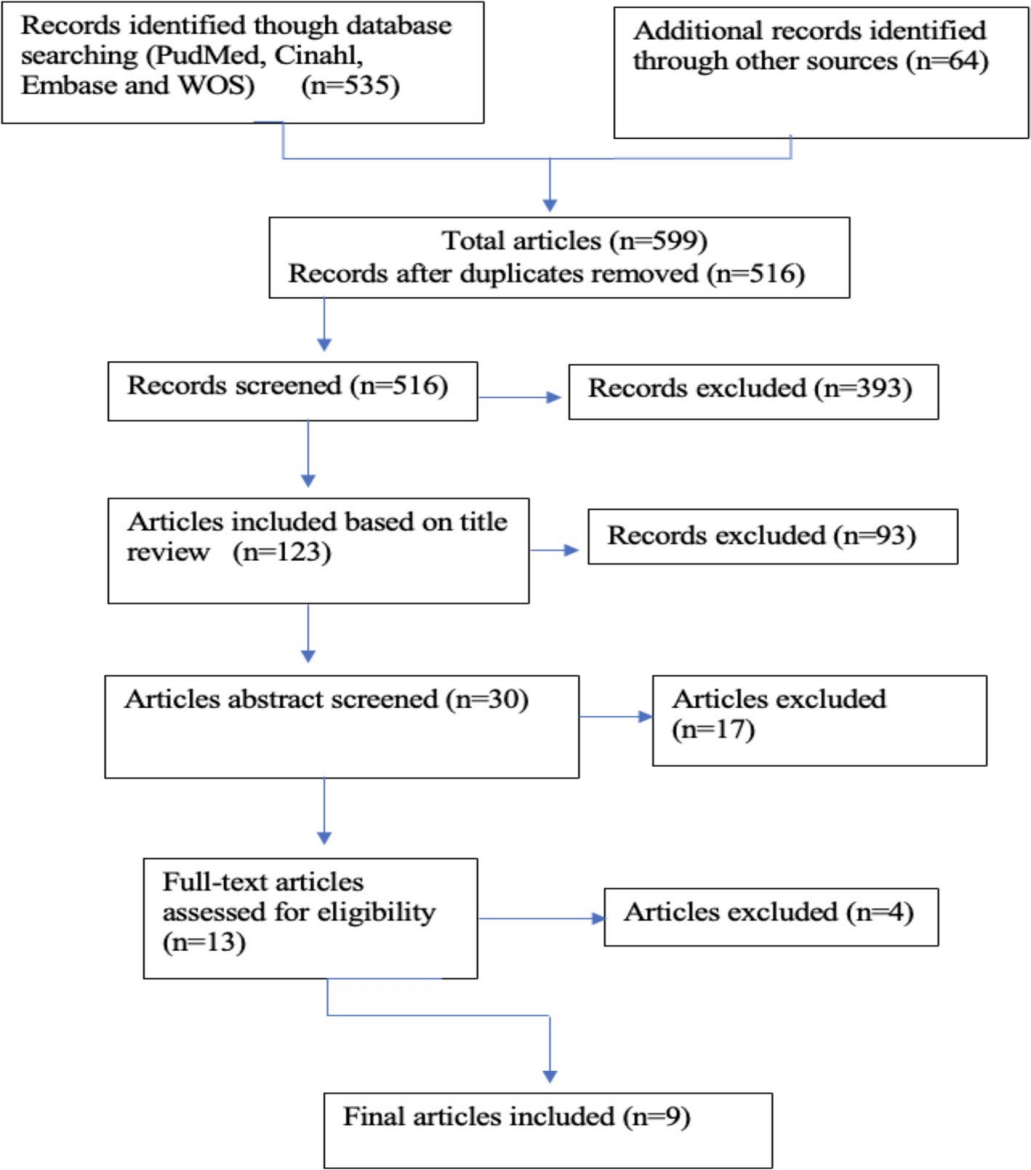
Flow chart of the review process

The inclusion criteria were studies with information on training in cultural competence, transculturality, transcultural care, and cultural care or similar terms in Spanish-speaking universities from Spain and Latin America The search was made by using Mesh terms and the Boolean operators: [AND], [OR], [NOT] were used to combine the Mesh terms. The peer-reviewed search retrieved 599 articles. Accordingly, duplicates were eliminated and articles with irrelevant titles and abstracts or that did not meet the inclusion criteria were removed from the review. In the next step, full texts of the articles were read and the ones that did not meet the criteria were excluded. After that, due to the lack of results, another complementary search was made in Google Scholar, which returned a large number of articles. Many of them were rejected because of the title and the summary and finally, only nine articles were considered. The representation of the literature search process is available in Fig. 1.

Later another search was carried out on gray literature. Firstly, contents, skills, and competencies from 47 Spanish universities were revised throughout the White Book of the Nursing Degree published by the National Agency for Quality Assessment and Accreditation in Spain (ANECA) which was created by the Spanish Ministry of Education to adapt the Nursing Program nationwide to the European Higher Education Space (EEES) [39]. Then similar Latin American official reports were summed up. Later the need to conduct a gap analysis came up and started by consulting the training programs of all the Spanish universities and the Latin American ones. All of them were searched for undergraduate and postgraduate programs, including master´s degrees and doctoral programs for nurses as well as research teams publishing on this topic. Subsequently, other training programs in the form of continuing professional development (CPD) offered by HEIs and educational organizations were explored. Additionally, we revised specific resources from the Boards of Nursing, Councils of Nurses, and Government organizations (at the national, regional, and local levels) such as the Repository TESEO that belongs to the Spanish Ministry of Education gathering all the doctoral thesis published by Spanish Universities; as well as other foundations, associations and scientific societies from Spain and Latin America and finally we decided to include some researchers’ insights and approaches related with the topic. Those researchers were four academics belonging to different Spanish Universities who participated as key informants and were identified through their publications on the topic and contacted via their public emails. They individually reviewed the data collected and assessed the process. A gap analysis was especially useful as a method of evaluation to identify in which organizations the educational gaps on cultural competence, transculturality, and cultural care had been covered until now, and the way it had been done.

#### Data extraction and analysis

The training programs of all the universities from Spain and Latin America have been consulted to extract in which universities these training in the form of subjects, courses or modules are taught, what type of learning form (compulsory/basic or optional), how much accreditation load they have (in ECTS format or in hours), what type of content includes, how the subject is distributed and taught (theoretical sessions, practical ones, simulation seminars, etc.) and how it is evaluated and then contrasted with the key academics selected. The results of the scoping review and gap analysis were analyzed and classified into two main categories: (A) Nursing Education in Spain and (B) Nursing Education in Latin America. The results were also shared with the key informants for feedback and validation. Within each category, other subcategories appeared informing on specific aspects.

## Results

### Nursing education in Spain

#### Cultural competence scientific production

*As shown in *Table 1*, a total of nine papers were included.* Evidence published shows that in Spain 63% of the nursing undergraduate programs offer training in transcultural care somehow, and of this percentage, only 17% consider those subjects as compulsory, the rest are either optional or these contents are part of other subjects [31]. The publications in scientific databases regarding this topic, in relation to Spanish-speaking countries and during the period of time of the search are scarce and usually refer to educational experiences and interventions conducted complementary to the official academic programs or in professional practice without any link with education.

**Table 1:** Extracted data

Author/s	Year	Theme	Origen	Participants	Type of study	Methods	Results
Granel N, Leyva-Moral JM, Morris J et al	2021	Student’s satisfaction and intercultural competence development from a short study abroad programs	Universities belong to the European Network of Nursing in Higher Education (ENNE)	Students of nursing from different European countries	Multicenter cross-sectional study	Implementation of a study abroad program	1 week abroad program. The results of the study showed that in a short period of time it was possible to get the students to acquire cultural competences and that these competences were still visible one year after the program
Antón-Solanas I, Tambo-Lizalde E, Hamam-Alcober N et al	2021	Nursing students’ experience of learning cultural competence	Spain, Belgium, Turkey and Portugal	Nursing students from universities in Spain, Belgium, Turkey and Portugal	Qualitative phenomenological study	Focus groups of 5 to 7 students	Only students from Spanish universities described an optional course in the fourth year of cultural competence. Most students describe cultural competence as part of some subject. They described insufficient and highly theoretical training
Sánchez Ojeda MA, Segura-Robles A, Gallardo-Virgil MA et al	2018	Enfermería Transcultural. Formación de los futuros profesionales de Enfermería en España	Spain	Study programs universities in Spain	Review	Nursing career study programs review at the universities of Spain	30 universities contain transcultural content or cultural diversity (63.3% of Nursing curricula). There are 18 compulsory ones, 9 are subjects of transculturality or similar and 9 have some related content. 13 are electives, 9 are subjects of transculturality or similar and 4 have some content
Gradellini C, Gómez-Cantarino S, Domineguez-Isabel et al	2021	Cultural Competence and Cultural Sensitivity Education in University Nursing Courses. A Scoping Review	Spain	Nursing programs that promote competence and cultural sensibility	Review	Review of the available literature	Due to the increase in the immigrant population, a specific model of cultural competence for nursing has been used in Spain, inspired by one carried out North America, at the University of Barcelona (Master)
Plaza del Pino et al	2022	Use of high-fidelity clinical simulation for the development of cultural competence of nursing students	Spain	Fourth-year nursing students	Qualitative descriptive study	High-fidelity simulation	Before the simulation scenario, there were insecurity, uncertainty, and cultural differences between the nursing students, with the main worry being the language barrier. They also said that they feel the need to improve their intercultural communication and cultural adaptation of the care in their training. After finishing the simulation, they showed positive aspects, aspects to be improved, learning acquired, and learning to be strengthened, highlighting the existing cultural differences and asking for training on cultural competence
Diego-Cordero et al	2022	Effects of an educational intervention on nursing students' attitudes towards gypsy women: A non-randomized controlled trial	Spain	Nursing student in university of Seville	Quasi-experimental study	An educational intervention among 40 nursing students in Spain based on the use of positive references in order to improve the students' attitude to- wards gypsy women receiving health care	The score in all the items that make up the Prejudicial Attitude Scale decreased after the test, which demonstrated that their attitudes had become less prejudiced. The Stereotype Content Model, the perception of the outgroup, and the stereotypes regarding the Roma population as “trustworthy” all improved showing a statistically significant difference between the periods (pre and post-educational intervention)
Ruidíaz K, Saldarriaga G, Gaviria P, Peinado	2019	Comparabilidad de un programa de Enfermería de Cartagena con programas internacionales: un análisis sistemático	Latin America	Nursing programs that promote competence and cultural sensitivity	Cross-sectional exploratory descriptive study	Bibliographic review and subsequent use of the manual colorimetry technique to review, refine and consolidate in order to highlight similar and comparative aspects of the study plans	Each continent has curricula that try to be consistent with the needs of each country, while in foreign universities they train nurses in the areas of innovation, environment, gender and health, as well as transculturality, the reference program offers other subjects such as general psychology, leadership and productivity, physical activity and innovation among others
Cedeño S, Rodríguez JI, Prieto DM et al	2021	Reflexiones sobre las bases conceptuales de la interculturalidad, las problemáticas y retos desde la formación de enfermería	Latin America	Nurses working in indigenous communities in your country	Qualitative study	semi-structured interviews	Countries such as Argentina, Chile, Colombia and Ecuador face the same difficulties in the poor training of nursing professionals in issues of multiculturalism for the health care of the different communities All these issues highlight the need to improve work in universities on intercultural health issues and for this to be cross-cutting at all levels
Cofré-González, CG, Alvarez-Cruces, DJ	2022	Fortalezas y debilidades sobre la incorporación de salud intercultural en la formación de enfermería	Latin America	Nursing professionals	Qualitative research with phenomenological approach	Intrinsic case study	The health professionals expressed that training in intercultural care is implicit or absent in the training itinerary, concluding that the need to promote intercultural encounters among clinical professionals is evident, as well as dynamic teaching methodologies where the contents are made more flexible and mainstreamed

Publications regarding cultural competence in Spanish-speaking practice settings were found though rejected as they were not related to training and education. In a study that took place in a Spanish university, a high-fidelity simulation was carried out with fourth-year undergraduate nursing students. The objective was to promote cultural competence with the immigrant population. The students were encouraged after the simulation to indicate positive aspects experienced, the items that should be improved, the learning acquired, and the learning that should be strengthened. It was highlighted that there are cultural differences they should be trained for. The use of high-fidelity simulation provided an important learning experience, which increased students´ comfort when dealing with patients of diverse cultural origins [40]. In another University, a quasi-experimental study was carried out after an intervention with nursing students. The intervention was based on the use of positive references on a minority population. The objective was to reduce prejudices and improve the quality of health care provided to the Roma ethnic group. Statistically significant results were obtained in the results in the reduction of prejudices measured through the Prejudicial Attitude Scale (PAT). The Stereotype Content Model (SCM), the perception of the outgroup, and the stereotypes regarding the Roma population as “trustworthy” all improved [41]. In another study carried out in 4 different countries, including Spain, only students from Spanish universities referred and described an optional course about cultural competence in the fourth year. Most of the participants in the study explained that cultural competence was part of some subjects and agreed that this training was insufficient and very theoretical [42]. Finally, another publication points out there is one Spanish University being part of the European Network of Nursing Higher Education (ENNE) which, together with other European HEIs, develops exchanges to promote transcultural care and cultural competence [43]. As part of such a positive experience, this university also developed a specific module in the form of a master's degree due to the increase in the immigrant population, to promote the acquisition of cultural competence [35].

#### Cultural competence and undergraduate nursing programs in Spain

Once having explored the 47 universities included in the ANECA White Book regarding the Quality Standards of the Nursing Degree in Spain, 29 had undergraduate training related somehow to transcultural care or cultural diversity. Of these 29 universities, the subject was compulsory in just 17 universities, being optional in 12 of them (Additional File 2). There is no consensus among the universities on the accreditation load that is given to this type of subject ranging from 3 to 6 ECTS (1 ECTS = 25 h approximately). Most of these universities teach these contents in theoretical sessions that are evaluated through written examinations of knowledge. These subjects are made up of theoretical and theoretical-practical training, including sometimes case-simulation seminars for the resolution of clinical cases, debates, discussions, or the analyses of articles and documentation that are normally provided by the teaching staff, and it is always taught in the classroom. Moreover, there is not a consensus on the contents of the theoretical training provided by the universities which are quite diverse, so that, in some faculties a small percentage of this training is allocated to general cultural aspects, giving more importance to other related topics such as gender, migration, vulnerable populations or minorities. There are some universities that divide the subject equally between multiculturalism and gender and a few invest more time in the concept of culture, ethnicity, and cultural knowledge. The cultural knowledge provided by the universities is quite diverse, while most universities approach it from a fairly general perspective, dedicating the subject to the knowledge and understanding of concepts such as “culture”, “interculturality”, “multiculturalism”, “globalization” or “cultural diversity”, others include in their contents the learning of culturally competent models such as the Purnell’s or Leininger's ones. Only one university has been identified that has summer courses for undergraduate students dedicated to cultural competence for students who need extra credits. Curiously none of the subjects explored among all the undergraduate programs in Spain stressed the importance of one’s own culture, its recognition, and cultural awareness. However, we identified official educational documents from the Spanish Ministry of Education that remark on the importance of cultural competence transversally within the nurses’ undergraduate program.

### Postgraduate nursing programs in Spain

#### Cultural competence and master degrees in Spain

After reviewing the study postgraduate programs of the Spanish universities included in the ANECA White Book on Nursing Education [39] we have been able to verify that training in Spain directly related to cultural competence, transcultural care, and even culture and health is limited when it comes to official master degrees. It is difficult to identify master's degrees in Spain aimed at directly training health professionals such as nurses in cultural competence. Most of the available ones are indirectly related or have a subject that deals with the topic in a diffuse way. A synthesis of the most relevant information on postgraduate training in cultural competence existing in the universities included in the ANECA White Book can be found in Additional File 3.

This review found some Master's degree programs are not directly focused on nurses, from the areas of social sciences and psychology, that some nurses interested in the topic had taken. Only two Spanish universities contain master´s degrees that directly deal with the topics of interest of this study. A total of 14 universities offer official master's degrees that allocate at least part of one subject to dealing with these issues. Curiously, these universities are coincident with the ones having more content in undergraduate programs and the professors and teachers are usually the same for undergraduate and postgraduate programs. Of these 14 universities, only two deal with cultural competence though this is not the central topic. Many of the master´s degrees that contain subjects and deal with culture are focused on qualitative research, existing only one university that dedicates an entire subject specifically to these topics [44]. There are other universities that although little, they deal with some multiculturalism in at least one subject. Two master's degrees in Public Health contain this type of subject, where health in multicultural contexts is included in one subject. Finally, two universities that contain master´s degrees whose training offer includes content regarding anthropology, indirectly related to this topic. None of the master´s degrees found allocates accreditation to practical healthcare training, which may be related to the fact that most of the master's degrees in Spain last one year. All the subjects are either theoretical-practical, understanding practices such as solving clinical cases in the classroom, discussions, and seminars, or they are exclusively theoretical. 

More detailed information can be found in supplementary file 3.

#### Cultural competence and doctoral programs and research

Only five universities in Spain have doctoral programs that directly include research teams whose names are at the same time directly linked with multicultural aspects in health, transcultural care, cultural competence, or other similar terms; however, the gap analysis allowed to identify other research teams working in those topics under generic terms and teams’ names and mostly linked with qualitative research and Nursing. It is worth noting that none of those five ones had a specific research pathway including the term cultural competence, however, there exist, among those five ones, terms such as culture, quality of life, cultural meditation, cultural diversity, immigration, ethnicity, or aging. Supplementary file 3 provides more detailed information.

Since the last reform of the Doctoral Programs at Spanish Universities, the doctoral programs related to Health Science are broader, with a multi-professional perspective, with varied pathways of research, where studies on different topics can be framed. Through the complementary consultation of the Spanish Doctoral Dissertations named TESEO, the small number of researchers working on these topics were easily identified through their own doctoral dissertations and the ones they had also mentored [45]. It is worth noting that these researchers mentoring doctoral dissertations, publishing articles, and contributing to their research teams are the same ones that usually teach the contents related to cultural competence and transcultural care in undergraduate and postgraduate programs in the different ways exposed until now. 

#### Cultural Competence and Non-official Training and other types of university nursing education

Only a couple of research teams that belong to HEIs have been identified for having been part of a few European Commission funded-projects based on multicultural nursing education, such as the GNURSESIM project and the HEALINT4ALL project. Again those are the same HEIs that have research teams, doctoral research pathways, experts’ publications, and doctoral dissertations mentored in relation to these topics. On the other hand, only one HEI has a university extension diploma in promoting interculturality to combat xenophobia, racism, Islamophobia, and anti-gypsyism (theoretical-practical) and a university extension diploma in culture and sport [46, 47].

A university that has an expert course in Spiritual Health was identified, and another one has a Senior Diploma in Culture, Science, Technology, and Society; however, all of them are not only focused on nurses, and specifically the last two ones are not taught in Spanish but in Catalonian language [48]. One fellowship and one course have been recently identified as part of one Foundation in Spain. There is also one Journal published in Spanish hosted in one Spanish University in relation to Culture and care and publishing most of the Spanish-speaking contributions [49]. 

### Continuing Professional Development (CPD)

Even though the academic informants mentioned CPD initiatives on cultural competence and transcultural care that once were active nationwide or in specific regions before 2017, during the search period it was not found any specific training on this topic was provided by the main CPD organizations [50, 51] and not even by the Council of Nurses and Boards of Nurses nationwide. Additionally, it was observed a paucity of proposals in geographic areas of Spain next to the boundaries where multiculturalism is part of daily life. Such is the case of Melilla and Ceuta on the south border with Africa, and the situation is similar in the principal cities that usually receive more foreigners and migration. The Andalusian Health Quality Agency in its quality certification program includes a range of quality standards related to cultural diversity, migration, transculturality, multiculturalism, interculturality, cultural diversity, cultural competence, and migration in the field of health care. However, only until 2005, more than 600 training activities were organized by health centers of the Andalusian public system related to these thematic areas [52] as it has happened in other Autonomous Communities in Spain.

### Nursing education in Latin America

The cases identified in Latin America are even more scarce compared with Spain due to the less number of Master's Degrees and Doctoral Programs available for Nurses as in some Latin American countries the profession is still not allowed to have such postgraduate academic level and their training is technical. However, specific particularities were in some Latin American countries: on the one hand, the fact that some undergraduate nursing programs emphasize the social and cultural aspects of specific ethnic groups and subcultures that are part of their own countries and have been co-living for years. However, that training in cultural competence is supposedly implicit though absent in the written training itinerary. On the other hand, some researchers and academics working in Latin America have completed their doctoral dissertations regarding cultural competence or transcultural care in Spain or associated with experts’ teams from Spain and are even nowadays linked with similar teams from Spain.

In 2019, a study was carried out that highlighted that universities outside Latin America pay more attention to subjects that allow the development of social aspects, studying multiculturalism, among other topics [53]. However, Latin American universities, like the Colombian ones, usually focus more on other subjects such as general psychology, leadership, productivity, physical activity, and innovation [54]. In countries like Chile, different higher education initiatives have been carried out regarding cultural aspects, especially in areas with a higher concentration of indigenous people, however, they have been approached and implemented in a very diverse way far from the professional training for nurses. There are many challenges to be achieved with respect to this intercultural approach, such as respect for diversity and human rights, participation, effective communication, and self-exploration of ethnocentric or culturally relativistic models that negatively affect intercultural dialogue [51]. Moreover, it was evident the need to promote intercultural encounters between clinical professionals as well as to develop teaching methodologies where the contents were transversal and flexible [55]. In Peru, the education in cultural competence for nurses is taught mainly from a contextual perspective, with limited training in interculturality focused on nursing interventions with cultural relevance, and the one that exists, often optional, is not always part of the knowledge contents of the nursing undergraduate program [56]. In Ecuador, the training of nursing professionals is also scarce both in issues related to cultural diversity and in addressing the linguistic barriers that health professionals have to face because Ecuador is a country with numerous subcultures, characterized for being multicultural and multiethnic. As part of their training, recent nursing graduates must complete a year of rural professional practice, where they work in health centers far from cities and where institutions lack resources. Although the topic of interculturality and ethnic groups is not part of the study programs, teachers must teach their students about these topics in some way, since recent graduates are required to take an exam in order to be hired by the Ecuadorian Health System, and in this exam, the "Comprehensive Family, Community, and Intercultural Health Care Model" is evaluated. Countries such as Argentina, Chile, and Colombia face the same difficulties in the limited training of nursing professionals in issues of multiculturalism for the health care of the different communities. All these issues highlight the need to improve work in universities on intercultural health issues and for this to be cross-cutting for all levels so that it is not only included within a subject such as anthropology but is worked on throughout the training period. Incorporating intercultural nursing into practice and its incorporation into care and care plans is also a challenge for Latin American countries [54].

## Discussion

In contrast to Spain, numerous studies have been conducted on nursing education regarding cultural competence and transcultural care in various countries, particularly English-speaking nations. In the case of Europe, most of them belong to the central and northern countries, especially the United Kingdom, though the majority come from outside the continent, especially the US [57–62].

Although some international studies have shown how the quality of health care can be compromised by both cultural and linguistic diversity [63–66] nursing educational programs in Spanish to provide professionals with suitable skills according to patients’ culture is low as it has been shown in this scoping review. Other studies highlight the importance of professionals’ initiatives towards facing complex situations for which they require training in cultural competence, aiming at making professionals feel more secure with their work so that patients receive quality and culturally competent care [67]. In line with these publications, this scoping review provides evidence about the scarce training on cultural competence promoted by HEIs in nursing undergraduate degrees and postgraduate programs as well as CPD, focusing on the types of programs, their contents, and evaluation assessments in Spanish-speaking countries. Therefore, in accordance with the results of this review, there are training programs in Spanish, including contents in undergraduate nursing degrees at some universities, though often being part of other courses or sometimes optional ones [31].

An existing barrier highlighted by previous research is that the vast majority of the training provided at different academic levels in relation to this topic is theoretical, as it can be also verified in the results of this scoping review and in other studies carried out [68]; however, there are studies that reveal that training in clinical practice is the most effective for developing knowledge, awareness, and safety among students [36]. Thus, Latin American nursing professionals have training that seems to be detached from professional practice, and this is contrary to what is necessary to train a professional, capable of acting and making real changes in their care practice [69]. Other forms of teaching, like exchanges between nursing students from different studies, have had positive results in the acquisition of cultural competence in Spain [40], coinciding with studies carried out in other countries such as one that took place among students from two universities, one from the US and the other from Thailand, where it was revealed that the experience was effective for students to gain confidence, increase their knowledge of different cultures as well as their cultural competence [58]. However, only one case conducted in Spain has been identified.

Despite the fact that university academics know and, in fact often feel concerned about how students can face the demands that a multicultural society requires from them [70], there are publications that pointed out that there are still situations to be resolved such as the variability in the study plans, that is to say, although there are subjects that in principle can have the same content and competencies, currently the training implementation is different depending on the university in which the subject is taught. Therefore, it is necessary to standardize the contents as well as teaching methods for nursing students from all cultures [11, 35] and these are results coincident with those of this scoping review. Other difficulties that could be found are the time invested in this training and the lack of reference standards [71], so it is also necessary to standardize the duration of the training [11].

In English-speaking countries, cultural competence has been considered a topic of great interest to the nursing profession for decades having scientific societies, university training, professional certifications, awards granted by states, specific hospital programs that address these areas, centers, journals, and institutions of both governmental and university research [30], while in Spanish-speaking countries, although it has become increasingly important, there are still many areas that need to be strengthened [54, 72] and the initiatives come from few researchers’ or academics’ teams settled in specific HEIs and Organizations. In fact, only one journal, one foundation, one recently founded fellowship, few courses and few research teams are active nowadays in Spain and to a lesser extent in Latin America. This may be because while multiculturalism has been present in the USA for many years, globalization has caused a rapid growth of cultural diversity in other areas such as Europe [73]. For this reason, it can be considered that for nurse educators at European universities, training in cultural competence is relatively new, so their own knowledge and skills are limited [61] and consequently this is also a barrier that must be overcome [30]. In addition, the current European initiatives have been developed in English which limits the participation of many experts and students.

### Limitations

HEIs programs and other official, and technical documents from different organizations have been limited to the period of time between 2017 and 2022 as it was understood that the topic is sensitive to the constant changes implemented in the HEIs’ curricula, and for such reason, we may have left out previous educational initiatives. However, this issue was overcome by including in the gap analysis the voice of key informants represented by academic experts that had been working on this topic even before 2017. The search has also been limited to Spanish and English languages without including publications in other ones as it was understood that the cases would also be language sensitive. Not many scientific publications have been found that meet the study inclusion criteria and that is why most of the data came from the gray literature selected from Spanish-speaking countries. It is also recommended that this review could be conducted prospectively to evaluate changes and improvements regarding the topic. Moreover, this scoping review highlights what type of training is available in Spanish-speaking countries for Nurses in these areas, but not if it is really useful for professionals in practice and generates perceived quality and satisfaction in populations as well as its return on investment.

## Conclusion

A global consensus in higher education and CPD on specific training for nurses becomes crucial.

This study shows that there is a lack of Global Consensus on Specific Training for Nurses and a lack of consensus in the load of ECTS related to cultural competence and transcultural care at different academic levels in the Spanish-speaking HEIs’ Nursing programs. Most of the universities count on transversal objectives to be achieved and evaluation items in relation to cultural competence within their practicums’ skills and competencies programs for undergraduate students though nearly unseen in the students’ evaluation reports. The training in many cases is blurred in other subjects and is quite diverse depending on the university where the degree is undertaken, even when it is a transversal competency that is part of numerous study programs. Furthermore, the implementation of initiatives in the Spanish-speaking HEIs are directly linked with particular researchers’ and educators’ interests and is much more present in Spain than in Latin America. The few pieces of evidence published that emphasize the importance of using didactic methods far from the classic theoretical sessions have not had any impact on the nurses’ training programs.

Postgraduate training (master's or doctoral level) in Spanish-speaking countries is heterogenous, mainly focusing on broad theoretical issues. Scientific production is limited to a small number of researchers, universities, or research groups. Important differences are found between Spain and Latin America that limit the progression of academic and advanced research regarding cultural competence.

It would be interesting to carry out studies in Spanish-speaking countries that assess whether study programs with clinical practices in cultural competence have an impact on the quality of care.

### Supplementary Information

**Additional file 1.** Search Strategy adapted to each database and the results.

**Additional file 2.** Undergraduate training in cultural competence in Spain.

**Additional file 3.** Postgraduate training in cultural competence in Spain.

## Data Availability

All data generated or analyzed during this study are included in this published article. (Additional file 1 -Search Strategy adapted to each database, Additional file 2 -Undergraduate training in cultural competence in Spain and Additional file 3 -Postgraduate training in cultural competence in Spain).

## Abbreviations

IOM: Organization for Migration
US: United States
TCNS: Transcultural Nursing Society
CTN: Certification in Transcultural Nursing
ANCC: American Nursing Credentialing Center
JTCN: Journal of Transcultural Nursing
UK: United Kingdom
ETNA: European Transcultural Nursing Association
ECTS: European Credit Transfers
HEIs: Higher Education Institutions
PRISMA-ScR: PRISMA Extension for scoping reviews
ANECA: National Agency for Quality Assessment and Accreditation in Spain
EEES: European Higher Education Space
CPD: Continuing professional development
PAT: Prejudicial Attitude Scale
SCM: Stereotype Content Model
ENNE: European Network of Nursing Higher Education
