# Supplementary material for: Exploring the education in cultural competence and transcultural care in Spanish for nurses and future nurses: a scoping review and gap analysis
Source: BMC Nurs. 2023 Sep 16;22:320. doi: 10.1186/s12912-023-01483-7 (PMC10504770; doi:10.1186/s12912-023-01483-7)
Supplement: Supplementary file 1 — Additional file 1. Search Strategy adapted to each database and the results. [file 12912_2023_1483_MOESM1_ESM.docx]

Additional file 1. Search Strategy adapted to each database and results.

| **Databases** | **Search strategy** | **Documents found** |
| --- | --- | --- |
| **PudMed** | Cultural nursing education AND (university OR practical training) AND Spain | 261 |
| **Cinahl** | Nursing education AND Cultural competency AND (University OR College OR higher education) | 47 |
| **Embase** | (‘cultural competency AND nursing education’ OR (cultural AND competent AND (‘nursing’/exp OR nursing) AND (‘education’/exp OR education))) AND (‘spain’/exp OR spain) | 11 |
|  | ‘cultural competency’ AND ‘university student’ | 10 |
|  | ('nursing education'/exp OR 'nursing education' OR (('nursing'/exp OR nursing) AND ('education'/exp OR education))) AND ('cultural competence'/exp OR 'cultural competence' OR (cultural AND ('competence'/exp OR competence))) AND ('university'/exp OR university OR 'college'/exp OR college OR 'higher education'/exp OR 'higher education' OR (higher AND ('education'/exp OR education))) AND ('latin america'/exp OR 'latin america' OR (latin AND ('america'/exp OR america))) | 11 |
| **WOS** | Transcultural nursing AND (universities OR practical training OR higher education) AND Spain. | 18 |
|  | Cultural competency AND Education AND (universities OR practical training OR higher education) | 173 |
| **Google Scholar** | Nursing education AND Cultural competency AND (Universities OR College OR higher education) and Spain and Latin-America | 10700 |
| **TOTAL** |  | 11231 |
