# Supplementary material for: Exploring the education in cultural competence and transcultural care in Spanish for nurses and future nurses: a scoping review and gap analysis
Source: BMC Nurs. 2023 Sep 16;22:320. doi: 10.1186/s12912-023-01483-7 (PMC10504770; doi:10.1186/s12912-023-01483-7)
Supplement: Supplementary file 2 — Additional file 2. Undergraduate training in cultural competence in Spain. [file 12912_2023_1483_MOESM2_ESM.pdf]

Additional File 2. Undergraduate training in cultural competence in Spain

University	Name of the subject	Type of subject	Credits	Grade	Note weighting	Part of the subject related to transculturality
U. of Almería	Género, Multiculturalidad y Salud.<br>(Gender, Multiculturality and Health)	Compulsory or basic subject	6 ECTS	1º	40% written theory test<br>40% written works<br>20% seminars or workshops	10 themes. 2 related to transculturality: Gender, Multiculturalism and Health: intrinsic relations and multiculturalism in the field of Health
U. Autónoma de Barcelona	Cultura, Sociedad y Salud.<br>(Culture, Society and Health.)	Compulsory or basic subject	6 ECTS	1º	10% self-evaluation<br>25% oral presentation of papers<br>40% written theory test<br>25% written works	All related topics (11 topics). For example Conceptual differences between race, ethnicity, culture, and community, among others. Ethnonursing or cultural competence: awareness, knowledge, skills, and need.
U. of Cádiz	Transculturalidad, Género y Salud<br>(Transculturality, Gender and Health)	Compulsory or basic subject	3 ECTS	2º	60% written theory test<br>20% written works<br>20% written practical course	Of 5 topics, 2 related to multiculturalism: Globalization and multiculturalism: diversity and cultural variability in the local and global spheres. Gender and multiculturalism and Immigration and health
U. of Granada.	Transculturalidad, Género y Salud<br>(Transculturality, Gender and Health)	Compulsory or basic subject	6 ECTS	1º	30% resolution of clinical cases<br>50% written theory test<br>20% seminars	3 Blocks. Related to globalization, multiculturalism, and gender. With topics such as Transcultural Care in the health-disease process: Transcultural Nursing.
U. of Jaén	Cultura, género y cuidados de salud<br>(Culture, gender and health care)	Compulsory or basic subject	6 ECTS	1º	70% written theory test<br>10% attendance<br>10% works, clinical cases or exercises<br>10% use of ICT tools, and laboratory practices	2 Modules. 1 focused on culture and the other focused on gender. In the block related to culture, you can find topics such as transcultural nursing care or the intercultural clinical interview.
U. of León.	Transculturalidad, salud y género<br>(Transculturality, Gender and Health)	Compulsory or basic subject	6 ECTS	1º	30% seminars and practices in classes<br>60% written theory test<br>10% attendance	5 theoretical blocks, 3 focused on culture and 2 focused on gender. In the block related to culture, you can find topics such as Characteristics that define a culture. Culture and ethnic definition. Gender Anthropology or The Nurse-User Relationship from the Socio-Cultural Perspective.
U. of Málaga	Cuidados Transculturales<br>(Cross-Cultural Care)	Compulsory or basic subject	6 ECTS	2º	70% written theory test<br>10% class participation<br>20% seminars/ practices	14 topics, all related to cultural competence and multiculturalism, for example: culture shock, transcultural nursing models, cultural competence in socio-sanitary care or communication with the culturally diverse patient.
U. of Vic	Cultura, Sociedad y Salud.<br>(Culture, Society and Health)	Compulsory or basic subject	6 ECTS	1º	40% written theory test<br>40% carrying out work and projects<br>10% the work done throughout the course<br>10% class participation	8 topics, 5 of them related to culture, like health and culture<br>Social and cultural factors that influence the health and illness of individuals, families, and communities, 1 related to gender, 1 to disability and another to social exclusion and marginalization

U. Europea of Madrid	Antropología cultural y de la salud.<br>(Cultural and health anthropology).	Compulsory or basic subject	3 ECTS	4º	30% written theory test<br>40% oral presentations of a project<br>30% observation of performance through text analysis.	3 blocks, all related to culture, for example: Culture and health in a global environment. Globalization and cultural diversity. Immigration and health.<br>Gender and health.
U. of Navarra	Antropología<br>(Anthropology)	Compulsory or basic subject	6 ECTS	1º	50% written theory test<br>20% written tests during the development of the subject<br>20% exhibition of knowledge in 3 practical classes<br>10% personal work research	The subject is focused on the knowledge of the person in relation to their origin and the meaning of their existence. Values to guide behavior.
U. of País Vasco	Antropología, Ética y Legislación<br>(Anthropology, Ethics and Legislation)	Compulsory or basic subject	6 ECTS	1º	75% written theory test<br>25% carrying out practices and transversal competence, based on solving exercises, cases or problems.	3 blocks. One related to legislation, another to society and anthropology and another more related to transculturality as well as gender and healthcare plurality (medicalization)
U. Ramón Llull	Antropología<br>(Anthropology)	Compulsory or basic subject	6 ECTS	2º	60% written theory test<br>20% individual written work<br>20% group written work	All issues related to transculturality but above all focused on suffering and death. For example: Death as a personal, cultural, social, religious event
U. Rey Juan Carlos	Enfermería Social, Género y Salud<br>(Social Nursing, Gender and Health)	Compulsory or basic subject	6 ECTS	1º	50% written theory test (70% of the correct answers to pass the test)<br>50% a job that can be done individually or in groups	4 blocks, 1 of them entirely dedicated to cultural care made up of 7 themes, the rest related to gender and its relationship with health, family and/or gender violence and socio-health care in situations of dependency, vulnerability and social exclusion.
U. Rovira i Virgili	Antropología y Ciudadanía<br>(Anthropology and Citizenship)	Compulsory or basic subject	6 ECTS	2º	40% theoretical oral exam<br>25% seminars<br>20% practice in which web pages, articles and documentation will be worked on and where it will be valued attendance and class participation<br>15% written works	7 thematic units and all related in some way to cultural competence, as well as qualitative research, cultural ethnocentrism and other topics related to transculturality.
U. San Pablo CEU Moncada	Antropología<br>(Anthropology)	Compulsory or basic subject	6 ECTS	1º	50% written theory test<br>25% partial theoretical exam	Little related to culture, almost all subjects of anthropology, knowledge of the human being both as a species and as a psycho-somatic-spiritual unit and bioethics

					20% written work<br>5% class participation	
U. of Valencia	Sociología, Género y Salud<br><br>(Sociology, Gender and Health)	Compulsory or basic subject	6 ECTS	1º	70% written theory test (35% sociology block and 35% health gender block)<br>30% individual written work	2 large blocks, one for sociology and the other for gender and health. The first block related to social issues, roles, status, health structures. Little related to transculturality
U. of Castilla la Mancha	Diversidad cultural y desigualdades sociales en salud<br>(Cultural diversity and social inequalities in health)	Compulsory or basic subject	6 ECTS	1º	70% written theory test<br>20% resolution of cases and/or problems through collaborative learning<br>10% class participation in discussions and exercise	4 topics, three of them related to cultural aspects, for example: “Social and cultural factors that shape identity processes and quality of life expectations” or “Social inequalities and their impact on the health/disease process. Social disadvantage, vulnerability and exclusion. Effects on the morbidity, mortality and health care” and the other related to gender and health.
U. of Alcalá	Enfermería ante la Transculturalidad y marginación<br>(Nursing in the face of Transculturality and marginalization)	Optional subject	6 ECTS	4º	Continuous evaluation, valuing the preparation and presentation of works, the resolution of cases and activities, the preparation of practical activities of the subject and especially the active and proactive participation in teaching activities will be taken into account. They will be assessed according to certain criteria, specifically 7 and each criterion will be assessed as excellent (outstanding), medium (remarkable), sufficient (approved) or insufficient (failed)	4 units, all related to transculturality, with units such as “Globalization, culture and multiculturalism”, “ Immigration, diversity and Health, Community”, “Analysis and community diagnosis” o “Tools for the social intervention in health”.
U. of Alicante.	Cultura de los cuidados, educación para el desarrollo y pensamiento crítico<br>(Culture of care, education for development and critical thinking)	Optional subject	6 ECTS	3º y 4º	60% carrying out work, class participation, seminars and group work.<br>40% written theory test	All topics related to culture. For example: “Culture of care, gender and humiliating practices: the case of FGM”, “Culture of care in ethnic minorities”, “Cultural competence and health care” or “Phenomenology of care and life experiences-health- disease-death”.
U. Pontificia Comillas, Madrid.	Enfermería Transcultural.<br>(Transcultural Nursing)	Optional subject	3 ECTS	4º	50% an open-response written test consisting of 3 questions to develop	5 modules, all related to culture and the last module focused on the practice of cultural care. With topics like “Culture concept”, “Transculturality-Interculturality”, “Nursing cultural models and theories” or “Approach to historical-cultural nursing analysis”.

					30% group work<br>20% the elaboration of 3 essays about a topic or a question in which they will have to make critical readings and reflect	
U. of Córdoba	Transculturalidad, Género y Salud.<br>(Transculturality, Gender and Health.)	Optional subject	6 ECTS	2º	50% written theory test<br>25% resolución supuestos prácticos/discusión<br>25% conducting scientific work	3 theoretical blocks. 2 related to transcultural care, diversity, cooperation and migration and the last one addressed to gender
U. Francisco Vitoria, Madrid.	Enfermería Transcultural<br>(Transcultural Nursing)	Optional subject	3 ECTS	4º	45% written theory test<br>30% group written work<br>15% student work in the classroom<br>10% attendance	8 blocks all related to culture in some way, for example: “Concept of culture, care and multiculturalism”, “Madeleine Leininger's theory” or “Social, economic and cultural determinants of health”
U. Internacional de Catalunya	Transcultural Nursing	Optional subject	3 ECTS	4º	30% written theory test<br>10% glossary<br>30% class participation, attendance, exercises and class work<br>30% group written assignments.	2 large blocks, one dedicated to transcultural nursing and the other dedicated to death and pain in diverse cultural environments. In the first one, you can find topics like “Madeleine Leininger's model: concept of health-disease, nursing care in this model” and within this it is related, for example, to decision-making within the different cultural values or communication with different social groups, families...
U. Las Palmas de Gran Canarias.	Atención a la Diversidad Cultural<br>(Attention to Cultural Diversity)	Optional subject	3 ECTS	2º	30% attendance and class participation in classroom and laboratory practices<br>20% oral presentation<br>20% individual and group works<br>30% written theory test	4 modules plus one introductory. All modules related to culture with topics such as: “Multiculturalism”, “Influence of culture on the perception and experience of the processes of health/illness/care” or “human oblations of diverse socio-cultural origin, present in Spain and in Canary Islands”.
U. Ramón Llull	Enfermería y multiculturalidad<br>(Nursing and multiculturalism)	Optional subject	3 ECTS	3º	60% individual written work<br>25% group written work<br>15% self-appraisal	3 blocks all related to culture and one of them especially focused on multicultural nursing. with topics such as: “Introduction to the concepts of: Culture, Interculturality, Multiculturalism”, “Social implications of multiculturalism” or “nursing care in multiculturalism: Intercultural communication, Most frequent health problems of the immigrant population or Larry Purnell's Cultural Competence Model”.
U. of Valladolid	Antropología de la salud, cuidados transculturales de enfermería<br>(Anthropology of health, transcultural nursing care)	Optional subject	3 ECTS	3º	70% written theory test<br>30% practices/exercises and presentation of results.	The subject consists of a single block related to cultures in health called “Cultures of Health: from techno-biological care to the integration of holistic care in society”.

U. of Alfonso X El Sabio	Asistencia Social y Marginalidad<br>(Social Assistance and Marginality)	Optional subject	4 ECTS		50% written theory test<br>30% resolution of practical cases<br>10% continuous assessment through a partial exam<br>10% class participation	9 topics, of which only one is related to culture and is rather focused on immigration.
U. Católica de Murcia.	Atención a Colectivos en Riesgo de Exclusión<br>(Attention to Groups at Risk of Exclusion)	Optional subject	6 ECTS	2º	70% theoretical practical exam and resolution of practical cases<br>30% class participation, carrying out work, resolution of practical cases and participation in discussion forums	3 units all related to marginalization and social exclusion, dealing with cultural diversity and interculturality in one of its topics.
U. of Murcia	Antropología de los cuidados y culturas de la salud.<br>(Anthropology of health care and cultures)	Optional subject	3 ECTS	3º	70% written theory test<br>20% written assignments and projects<br>10% public presentation of works	4 topics, 2 related to social anthropology such as the health-disease concept, the third related to cultures, dealing with various areas such as childbirth, parenting, care for the mentally ill or care for the elderly and studying cross-cultural models such as Leininger and a final topic focused on research
