# Supplementary material for: Exploring the education in cultural competence and transcultural care in Spanish for nurses and future nurses: a scoping review and gap analysis
Source: BMC Nurs. 2023 Sep 16;22:320. doi: 10.1186/s12912-023-01483-7 (PMC10504770; doi:10.1186/s12912-023-01483-7)
Supplement: Supplementary file 3 — Additional file 3. Postgraduate training in cultural competence in Spain. [file 12912_2023_1483_MOESM3_ESM.pdf]

Additional File 3. Postgraduate training in cultural competence in Spain

*** : DIRECTLY ADDRESSES TRANSCULTURAL ISSUES**
#:IT HAS NO RELATIONSHIP WITH HEALTH
(X)**: Disposable BECAUSE NO CULTURAL OR OTHER REASONS**
~: INCLUDES SOME TRANSCULTURALITY ISSUES

University	Official master's degrees	Doctorates	Own university education	Another formation
U. of Alcalá	~Máster universitario en investigación en ciencias sociosanitarias.<br>~(Master's degree in research in socio-health sciences)<br>(Subject: Inequality and health.<br>1 SEMINAR OF 2 HOURS on the influence of culture and gender on health).	(X)Programa epidemiología y salud pública. Línea de investigación: Determinantes socioeconómicos y desigualdades en salud. Investigación en servicios de salud<br><br>(X)(Epidemiology and Public Health Program. Research line: Socioeconomic determinants and health inequalities. Health services research)	Not available	Not available
U. of Alfonso X El Sabio	Not available	Not available	Not available	Not available
U. of Alicante	*Máster Universitario en Investigación en Ciencias de la Salud.<br>Asignatura: Historia y cultura de los cuidados (6 ECTS)<br><br>*(Master's Degree in Research in Health Sciences.<br>Subject: History and culture of care (6 ECTS))	*Programa en ciencias de la salud. Líneas de investigación:<br>-Antropología de los cuidados<br>-Innovación y mejora en cuidados de salud<br>-Desigualdades en salud según: clase social, género y etnia<br>-Inmigración y Salud<br>-Población y salud: estadísticas sanitarias, análisis de la mortalidad, inmigración, etnicidad y envejecimiento<br>-Enfermería y salud comunitaria.<br><br>*(Program in health sciences. Lines of investigation:<br>-Anthropology of care<br>-Innovation and improvement in health care<br>-Inequalities in health according to: social class, gender and ethnicity<br>-Immigration and Health<br>-Population and health: health statistics, analysis of mortality, immigration, ethnicity and aging<br>-Nursing and community health)	Not available	Not available
U. of Almería	Not available	~Programa de salud, psicología y psiquiatría. Líneas de investigación:<br>-Avances e innovación en enfermería	Not available	Not available

		~(Health, psychology and psychiatry program. Lines of investigation:<br>-Advances and innovation in nursing)		
U. Autónoma of Barcelona	~Máster Universitario en Innovación Enfermera Aplicada a la Vulnerabilidad y a la Salud. Asignatura: Fundamentos Teóricos de la Vulnerabilidad (6 ECTS), 3 bloques uno de ellos relacionado con inmigración y multiculturalidad y Grupos Vulnerables en la Edad Adulta y la Vejez (9 ECTS), 4 bloques en uno se incluyen temas relacionados con inmigración y grupos minoritarios.<br>~University Master's Degree in Nursing Innovation Applied to Vulnerability and Health. Subject: Theoretical Foundations of Vulnerability (6 ECTS), 3 blocks one of them related to immigration and multiculturalism and Vulnerable Groups in Adulthood and Old Age (9 ECTS), 4 blocks in one include topics related to immigration and minority groups.	Not available	(X)Máster propio en salud internacional y cooperación<br><br>(X)(Master's degree in international health and cooperation)	GRIVIS: Grup de Recerca Infemera en Vulnerabilitat i Salut. It is a research group recognized by the Catalan university quality agency.
U. Autónoma of Madrid	*Máster Universitario en Investigación y Cuidados de Enfermería en Poblaciones Vulnerables. Asignatura: Fenómenos migratorios y exclusión social (3 ECTS). 3 bloques, uno de ellos aborda la competencia cultural.<br>*(Master's Degree in Research and Nursing Care in Vulnerable Populations.<br>Subject: Migratory phenomena and social exclusion (3 ECTS). 3 blocks, one of them deals with cultural competence).	~Programa de epidemiología y salud pública. Líneas:<br>-Determinantes socioeconómicos y desigualdades en salud.<br>-Investigación en servicios de salud<br>~(Epidemiology and Public Health Program. Lines:<br>-Socioeconomic determinants and inequalities in health.<br>-Research in health services).	Not available	Not available
U. of Cádiz	Not available	Programa en Ciencias de la Salud. Líneas de investigación:<br>-Psicología, educación y salud<br>(Program in Health Sciences. Lines of investigation:<br>-Psychology, education and health).	Not available	Not available
U. of Cantabria	~Máster en Investigación en Cuidados de Salud.<br>Asignatura:<br>Aspecto psicosociales y antropológicos (3 ECTS). Un tema dedicado a antropología de los	(X)Programa en Medicina y Ciencias de la Salud.<br>Líneas de investigación:<br>-Epidemiología, salud pública y ambiental y atención primaria.<br>(X)(Program in Medicine and Health Sciences.<br>Lines of investigation:	Not available	Not available

	cuidados, otro determinantes de salud, modelo biopsicosocial.<br>~(Master in Health Care Research. Subject: Psychosocial and anthropological aspects (3 ECTS). A theme dedicated to the anthropology of care, other determinants of health, biopsychosocial model).	-Epidemiology, public and environmental health and primary care.)		
U. of Cardenal Herrera CEU Moncada of Valencia	Not available	Not available	Not available	Not available
U. of Castilla la Mancha	Not available	Programa Ciencias de la Salud<br>(Health Sciences Program)	Not available	Not available
U. Católica San Antonio of Murcia	Not available	~Programa de Ciencias de la Salud. Línea de investigación:<br>-Cuidados de salud<br>~(Health Sciences Program. Line of research:<br>-Healthcare)	Not available	Sección I+D. Grupos de investigación:<br>-EVICUSA: Equipo de Valoración e Intervención en Cuidados de Salud<br>-Pensamientos y lenguajes enfermeros en Contexto Social<br><br>(R&D section. Investigation groups:<br>-EVICUSA: Health Care Assessment and Intervention Team<br>-Nursing Thoughts and Languages in Social Context)
U. Pontificia Comillas of Madrid	Not available	~Programa de salud, bienestar y bioética. Línea de investigación:<br>-Humanización en la asistencia y la docencia.<br>-Cuidados de salud y sociedad<br>~(Health, wellness and bioethics program. Line of research:<br>-Humanization in attendance and teaching.<br>-Health care and society)	Not available	Not available
U. Complutense of Madrid	#Máster en Estudios Interculturales europeos. Enfoque multicultural, trata globalización, diversidad cultural en Europa, estrategias de integración.<br>#Master in European Intercultural Studies. Multicultural approach, deals with globalization, cultural diversity in Europe, integration strategies (not directly related to health).	~Programa cuidados en salud. Línea de investigación:<br>-Investigación en cuidados<br>~(Health care program. Line of research:<br>-Research in care<br><br>In the area of sociology and anthropology programs, if there are lines of research related to culture	#*Máster propio en gestión cultural Internacional e Innovación Social<br>*Own Master's degree in International Cultural Management and Social Innovation Focused on economy, project	Not available

			development and education	
U. of Córdoba	Not available	~Programa en biomedicina. Líneas de investigación:<br>-Equipo de investigación multidisciplinar en atención primaria y comunitaria, y en cuidados integrales.<br>~(Program in biomedicine. Lines of investigation:<br>-Multidisciplinary research team in primary and community care, and in comprehensive care).	Not available	Not available
U. of La Coruña	*Máster universitario en asistencia e investigación sanitaria. Asignaturas:<br>-Investigación en Ocupación Humana y las Múltiples Diversidades.<br>-Investigación en Ocupación Humana y Determinantes Sociales de la Salud.<br><br>*(Master's degree in health care and research. Subjects:<br>-Research on Human Occupation and Multiple Diversities<br>-Research on Human Occupation and Social Determinants of Health.)	~Programa en ciencias de la salud. Líneas de investigación:<br>-Epidemiología y Salud Pública<br><br>~(Program in health sciences. Lines of investigation:<br>-Epidemiology and Public Health)<br><br>This line is part of a research group within this group, 3 sub-lines are investigated, one of which is qualitative research in health care.	Not available	Not available
U. Europea of Madrid	~Máster en Salud Pública (Online). Módulo: Acciones de salud pública en situaciones de vulnerabilidad: Salud en contextos multiculturales (subtema)<br><br>~Master in Public Health (Online). Module: Public health actions in situations of vulnerability: Health in multicultural contexts (subtopic)	~Programa en biomedicina y ciencias de la salud.<br>Líneas de investigación:<br>-Cuidados avanzados en enfermería<br><br>~(Program in biomedicine and health sciences.<br>Lines of investigation:<br>-Advanced nursing care)	Not available	Not available
U. of Extremadura	Not available	It has not been found (does not allow access to lines of investigation)	Not available	Not available
U. Francisco de Vitoria of Madrid	Not available	~Programa de educación médica, innovación docente y humanización y salud<br>~(Medical education, teaching innovation and humanization and health program)	Not available	Not available
U. of Girona	*Máster en Promoción de la Salud.	*Programa en psicología, salud y calidad de vida.<br>Líneas de investigación:	#Máster propio en diversidad religiosa:	Not available

	Asignatura: Antropología (3 ECTS). Teórico-práctica.<br><br>*Master in Health Promotion.<br>Subject: Anthropology (3 ECTS). Theoretical-practical.	- Subjetividades, discursos y contextos. Incluye varios puntos entre ellos:<br>-Etnografía: investigación sobre prácticas culturales e imaginarios colectivos<br>-Subjetividades, prácticas discursivas y relaciones de poder en la ciencia, la cultura y las interacciones cotidianas.<br>-Ciencia, construcción de la diferencia y sociedad.<br>-Envejecimiento, discapacidad, cultura y salud.<br><br>*(Program in psychology, health and quality of life.<br>Lines of investigation:<br>- Subjectivities, discourses and contexts. It includes several points among them:<br>-Ethnography: research on cultural practices and collective imaginaries<br>-Subjectivities, discursive practices and power relations in science, culture and everyday interactions.<br>-Science, construction of difference and society.<br>-Aging, disability, culture and health)	pensamiento, cultura y gobernanza.<br><br>#(Own master's degree in religious diversity: thought, culture and governance.)<br>(Not directly related to health).	
U. of Granada	Not available	~Programa en Medicina Clínica y Salud Pública.<br>Línea de investigación:<br>-Investigación en enfermería, fisioterapia y terapia ocupacional<br><br>~(Program in Clinical Medicine and Public Health.<br>Line of research:<br>-Research in nursing, physiotherapy and occupational therapy)	Not available	*Equipo y área de investigación Aspectos psicosociales y transculturales de la salud y la enfermedad.<br>(Team and research area Psychosocial and cross-cultural aspects of health and disease)
U. of Huelva	Not available	~Programa en ciencias de la salud. Líneas de investigación:<br>-La salud y sus determinantes<br>-Instrumentos y diagnóstico en salud<br><br>~(Program in health sciences. Lines of investigation:<br>-Health and its determinants<br>-Instruments and diagnosis in health)	Not available	Not available
U. of les Illes Balears	Not available	(X)Programa en Salud Global, Servicios de Salud y Atención socio-sanitaria.<br>Líneas de investigación:<br>- Evidencias en cuidados de salud y atención socio-sanitaria.	Not available	#Investigation groups. Program called cuidados, cronicidad y evidencias en salud (care, chronicity and evidence in health) that contains a line of research called: Transformación de los entornos clínicos, implementación del conocimiento, mejora de la práctica clínica y humanización (Transformation of

		-Epidemiología, salud pública global y desigualdades en salud.<br>(X)(Program in Global Health, Health Services and Social and Health Care.<br>Lines of investigation:<br>- Evidence in health care and socio-health care.<br>-Epidemiology, global public health and health inequalities).		clinical environments, implementation of knowledge, improvement of clinical practice and humanization.)
U. Internacional de Catalunya	Not available	#Programa en ciencias de la salud.<br>Líneas de investigación:<br>-Evaluación de los determinantes de salud<br><br>#(Program in health sciences.<br>Lines of investigation:<br>-Assessment of health determinants)	Not available	Not available
U. of Jaén	Not available	(X)Programa de doctorado interuniversitario en ciencias de la salud. Líneas de investigación;<br>-Procedimientos clínicos, intervenciones y resultados en salud.<br>-Género y determinantes sociales en salud.<br>-Epidemiología, salud pública y gestión sanitaria.<br>(X)(Interuniversity doctoral program in health sciences. Lines of investigation;<br>-Clinical procedures, interventions and health outcomes.<br>-Gender and social determinants in health.<br>-Epidemiology, public health and health management.)	Not available	#Diploma de extensión universitario en promoción de la interculturalidad para combatir la xenofobia, el racismo, la islamofobia y el antigitanismo (teórico-práctico)<br>- Diploma de extensión universitaria cultura y deporte<br><br>#(University extension diploma in promotion of interculturality to combat xenophobia, racism, Islamophobia and antigypsyism (theoretical-practical)<br>- Diploma of university extension culture and sport)<br><br>*Both for UJ students
U. of La Laguna	*Máster universitario en Investigación, Gestión y Calidad en Cuidados para la Salud.<br>Asignatura: Cuidados de Salud (6 ECTS). Contenido en transculturalidad.<br><br>*(Master's degree in Research, Management and Quality in Health Care.<br>Subject: Health Care (6 ECTS). Cross-cultural content.)	~Programa de ciencias de la salud.<br>Línea de investigación:<br>-Salud Pública, Salud Mental y Cuidados para la salud<br><br>~(Health sciences program.<br>Line of research:<br>-Public Health, Mental Health and Health Care)	Not available	Not available
U. of La Rioja	Not available	(X)Programa en ciencias biomédicas y biotecnológicas<br>(X)(Program in biomedical and biotechnological sciences)	Not available	Not available

U. of Las Palmas de Gran Canarias	Not available	(X)Programa en Investigación aplicada a las Ciencias Sanitarias<br><br>(X)(Research Program Applied to Health Sciences)	Not available	Not available
U. of León	#Master en cultura y pensamiento. Europa: tradición y pervivencia.<br>#Master in culture and thought. Europe: tradition and survival. (Belongs to arts and humanities not to Health Sciences)	Not available	Not available	Not available
U. of LLeida	Not available	~Programa Salud.<br>Líneas de investigación:<br>- Enfermería y salud<br><br>(X)Programa cronicidad, dependencia y salud en la comunidad.<br>Líneas de investigación:<br>-Cronicidad y atención a personas frágiles y envejecidas<br>-Salud en la comunidad<br>-Salud de la mujer<br><br>#*Programa Territorio, patrimonio y cultura<br><br>(X) (Health Program.<br>Lines of investigation:<br>- Nursing and health<br><br>Chronicity, dependency and health program in the community.<br>Lines of investigation:<br>-Chronicity and care for frail and elderly people<br>-Health in the community<br>-Women's health<br><br>#*Territory, heritage and culture program (does not belong to health sciences))	Not available	Diplomado/a sénior en cultura, ciencia, tecnología y Sociedad.<br><br>Senior Diploma in Culture, Science, Technology and Society.<br>On-site, taught in Catalan, 120 credits. It touches many topics, such as ecological, nutritional, geographical, philosophical, as well as health. Closely related to Catalonia.

U. of Málaga	*Máster en Salud Internacional. Asignatura:<br>-Investigación aplicada al ámbito cultural (6 ECTS). Incluye formación directa en competencia cultural. Teórica-práctica.<br>-Globalización y Salud.<br>*(Master in International Health.Subject:<br>-Research applied to the cultural field (6 ECTS). Includes direct training in cultural competence. Theoretical-practical.<br>-Globalization and Health)	(X)Programa de ciencias de la salud.<br>Línea de investigación:<br>-Metodología y herramientas de investigación en la valoración de resultados en salud<br><br>(X)(Health sciences program.<br>Line of research:<br>-Methodology and research tools in the assessment of health outcomes)	Not available	
U. of Murcia	(X)Máster en Salud Pública. Asignatura: Promoción de la salud (3 ECTS).<br><br>(X)Master in Public Health. Subject: Health promotion (3 ECTS). Addresses issues of immigration and social inequalities in health	~Programa en Ciencias de la Salud. Equipo investigador (Aparato Locomotor y radiología, enfermería, estomatología y odontología, fisioterapia y gestión de la calidad)<br>Líneas de investigación<br>-Cronicidad, dependencia y cuidados basados en la evidencia<br>~-Acceso a la salud en población migrante y el colectivo LGBTQ<br>~-Enfermería<br>(X)-Salud Pública y Epidemiología<br><br>~(Program in Health Sciences. Research team (Locomotor System and radiology, nursing, stomatology and dentistry, physiotherapy and quality management)<br>Lines of investigation<br>-Chronicity, dependency and evidence-based care<br>~-Access to health in the migrant population and the LGBTQ community<br>~-Nursing<br>(X)-Public Health and Epidemiology)	Not available	Not available
U. of Navarra	Not available	~Programa en ciencias de la enfermería.<br>Líneas de investigación:<br>-Innovación para un cuidado centrado en la persona.<br>-Innovación para la promoción de la salud familiar y comunitaria.<br>~(Program in nursing sciences.<br>Lines of investigation:<br>-Innovation for person-centered care.	Not available	Not available

		-Innovation for the promotion of family and community health).		
U. of Oviedo	Not available	~Programa en ciencias de la salud.<br>~(Program in health sciences.)	Not available	#*Experto universitario en Interculturalidad, justicia y cambio global.<br><br>#*University expert in interculturality, justice and global change. (Not related to health).
U. of País Vasco	Not available	~Programa en Salud Pública.<br>~(Program in health sciences.)	(X)Máster propio de salud y salud comunitaria. Asignaturas: determinantes de la salud, desigualdades sociales.<br>(X)(Master's degree in health and community health. Subjects: determinants of health, social inequalities)	Not available
U. Pública of Navarra	*Máster en salud pública.<br>Asignatura: Salud, cultura y sociedad.<br>(Master in public health.<br>*Subject: Health, culture and society. (4.5 ECTS).	~Programa doctorado en ciencias de la salud.<br>Línea de investigación:<br>-Salud pública, enfermería y medicina<br><br>~(Doctoral program in health sciences.<br>Line of research:<br>-Public health, nursing and medicine)	Not available	Not available
U. Ramón LLull	Not available	*Programa en Salud Bienestar y bioética.<br>Línea de investigación:<br>-Global health, gender and society<br>-Exploración de los determinantes sociales, el contexto cultural con perspectiva de género y sus impactos potenciales sobre la salud de las personas.<br><br>*(Program in Health Well-being and bioethics.<br>Line of research:<br>-Global health, gender and society	*Máster propio en espiritual transcultural.<br>Máster en diálogo interreligioso, ecuménico y cultural.<br>*(Master's degree in transcultural spirituality. It	#Curso Experto Universitario en Salud Espiritual Una base psicológica.<br>#(University Expert Course in Spiritual Health A psychological basis)<br><br>Taught in Catalan (15 ECTS)

		-Exploration of social determinants, the cultural context with a gender perspective and its potential impacts on people's health).	includes immersion practices in communities or spiritual centers, national or international. Master in interreligious, ecumenical and cultural dialogue.)	
U. Rey Juan Carlos	*Máster oficial en bioética<br>Asignatura: antropología.<br>*(Official master's degree in bioethics (face-to-face). Subject: Anthropology).	~Programa en ciencias de la salud.<br>Programa de doctorado en epidemiología y salud pública (Interuniversitario).<br>Líneas de investigación:<br>-Determinantes socioeconómicos y desigualdades en salud<br>-Investigación en servicios de salud<br><br>~(Program in Health Sciences.<br>Doctoral program in epidemiology and public health (Interuniversity).<br>Lines of investigation:<br>-Socioeconomic determinants and health inequalities<br>-Research in health services)	Not available	Not available
U. Rovira i Virgili	*Máster en antropología médica y salud global.<br>Asignaturas:<br>-Alimentación, salud y cultural<br>-Antropología y salud global<br>-Antropología y salud mental<br>-Salud y sociedad en la historia de occidente<br><br>*Máster en Ciencias de la enfermería.<br>Asignatura:<br>-Investigación en cultura y cuidado.<br><br>*Master in medical anthropology and global health. Subjects:<br>-Food, health and culture<br>-Anthropology and global health<br>-Anthropology and mental health<br>-Health and society in Western history<br><br>*Master of Science in Nursing. Subject:	(X)Programa en enfermería y salud. Línea de investigación:<br>-Dimensiones del cuidado: género, familia y comunidad.<br><br>(X)(Program in nursing and health. Line of research:<br>-Dimensions of care: gender, family and community)	Not available	Not available

	-Research in culture and care (3 ECTS). Theoretical-practical.			
U. of Salamanca	Not available	*Programa de salud y desarrollo en los trópicos.<br>Líneas de investigación:<br>-Evaluación, interpretación y tratamiento fisioterápico del dolor crónico en diferentes entornos culturales.<br>-Antropología Médica y Epidemiología cultural<br>-Mediación cultural. Género y diversidad cultural<br>-Rehabilitación psicosocial y psicopatología transcultural.<br><br>*(Health and development program in the tropics.<br>Lines of investigation:<br>-Evaluation, interpretation and physiotherapy treatment of chronic pain in different cultural environments.<br>-Medical Anthropology and Cultural Epidemiology<br>-Cultural mediation. Gender and cultural diversity<br>-Psychosocial rehabilitation and transcultural psychopathology).	Not available	Not available
U. of San Pablo CEU Moncada de Madrid	Not available	Not available	Not available	Not available
U. Santiago of Compostela	Not available	(X)Programa de doctorado en epidemiología y salud pública.<br><br>(X)(Doctoral program in epidemiology and public health)	Not available	(X)*A summer course was offered between June 16 and 18, 2021 in cultural competency for care and sustainable development goals. It was a 15 hour course.
U. of Sevilla	*Máster Universitario en Migraciones Internacionales, Salud y Bienestar: Modelos y Estrategias de Intervención.<br>(X)Máster Universitario en Nuevas tendencias asistenciales en Ciencias de la Salud. Asignaturas: Estrategias de Promoción de la Salud en un contexto Multicultural (4 ECTS).<br>#Enfocada a Promoción de la Salud y Educación para la Salud.<br><br>*(Master's Degree in International Migration, Health and Well-being: Models and Intervention Strategies. All subjects related to multiculturalism.<br>(X)Master's Degree in New Care Trends in Health Sciences. Subjects: Health Promotion Strategies in a Multicultural Context (4 ECTS).<br>#Focused on health promotion and Education for health).	(X)Programa de ciencias de la salud (Interuniversitario). Línea de investigación:<br>-Género y determinantes sociales en salud.<br><br>(X)(Health Sciences Program (Interuniversity). Line of research:<br>-Gender and social determinants in health.)	Not available	Not available

U. of Valencia	Not available	~Programa de doctorado en enfermería clínica y comunitaria.<br>Línea de investigación:<br>-Salud de la población<br><br>~(Doctoral program in clinical and community nursing.<br>Line of research:<br>-Population health)	Not available	Not available
U. of Valladolid	Not available	(X)Programa en investigación en ciencias de la salud.<br>Líneas de investigación:<br>-Grupo de investigación en valoración e intervención multidisciplinar en atención sanitaria y estilos de vida sostenibles.<br><br>(X)(Research program in health sciences.<br>Lines of investigation:<br>-Research group on multidisciplinary assessment and intervention in health care and sustainable lifestyles).	Not available	Not available
U. of Vic	Not available	*Programa de doctorado en estudios de género: cultura, sociedades y políticas.<br>Líneas de investigación:<br>-Salud.<br>(X)Programa de doctorado en cuidados integrales y servicios de salud.<br>Líneas de investigación:<br>-Cronicidad, dependencia y salud en la comunidad<br>-Servicios de salud y aplicación de resultados en salud.<br>-Políticas sociales y prácticas en salud (sub-línea: determinantes sociales y desigualdades en salud o políticas y prácticas en salud).<br><br>*(Doctoral program in gender studies: culture, societies and politics.<br>Lines of investigation:<br>-Health.<br>(X)Doctoral program in comprehensive care and health services.<br>Lines of investigation:<br>-Chronicity, dependency and health in the community<br>-Health services and application of health results.	Not available	Not available

		-Social policies and practices in health (sub-line: social determinants and inequalities in health or policies and practices in health).		
U. of Zaragoza	*Máster Universitario en Iniciación a la Investigación en Ciencias de la Enfermería. Asignatura:<br>-Cuidados ecológicos y holísticos<br>-Bases documentales y legales, económicas, sociales y culturales de los cuidados a nivel europeo.<br><br>*(Master's Degree in Initiation to Research in Nursing Sciences. Subject:<br>-Ecological and holistic care (6 ECTS). 11 modules and one is cross-cultural nursing. Theoretical-practical.<br>-Documentary and legal, economic, social and cultural bases of care at a European level. (6 ECTS). 11 topics, one of them is cultural diversity in health. Theoretical-practical).	(X)Programa en Ciencias de la Salud y del deporte.<br>Línea de investigación:<br>-Actividad física, salud y calidad de vida.<br><br>(X)(Program in Health and Sports Sciences.<br>Line of research:<br>-Physical activity, health and quality of life).	Not available	Not available
U. of Barcelona	*Máster en estudios avanzados en exclusión social. Asignatura:<br>-Cultura, arte y sociedad inclusiva.<br><br>*Máster de Metodología de la Investigación Aplicada a los cuidados enfermeros. Asignatura:<br>-Género y desigualdades en la Investigación.<br><br>#Máster en Intervención psicosocial. Asignatura:<br>-Cultura, inclusión y exclusión social. (10 ECTS).<br><br>*(Master in advanced studies in social exclusion. Subject:<br>-Culture, art and inclusive society (8 ECTS).<br><br>*Master's Degree in Research Methodology Applied to Nursing Care. Subject:<br>-Gender and inequalities in Research (3 ECTS). She talks about cultural inequalities but adapted to women. Theoretical-practical.<br><br>#Master in psychosocial intervention. Subject:	(X)Programa: enfermería y salud. Líneas de investigación:<br>-Dimensiones del cuidado: género, familia y sociedad<br><br>(X)(Program: nursing and health. Lines of investigation:<br>-Dimensions of care: gender, family and society).	Not available	Not available

	-Culture, inclusion and social exclusion. (10 ECTS). Dedicated to culture, migration and social inclusion and exclusion. (Not related to health). Theoretical-practical)			
